# Exogenous and Endogenous Phosphoethanolamine Transferases Differently Affect Colistin Resistance and Fitness in *Pseudomonas aeruginosa*

**DOI:** 10.3389/fmicb.2021.778968

**Published:** 2021-10-27

**Authors:** Matteo Cervoni, Alessandra Lo Sciuto, Chiara Bianchini, Carmine Mancone, Francesco Imperi

**Affiliations:** ^1^Department of Science, Roma Tre University, Rome, Italy; ^2^Department of Molecular Medicine, Sapienza University of Rome, Rome, Italy; ^3^IRCCS Fondazione Santa Lucia, Rome, Italy

**Keywords:** cell envelope, EptA, lipid A, MCR, phosphoethanolamine (PEtN), polymyxin, 4-amino-4-deoxy-L-arabinose

## Abstract

Colistin represents a last-line treatment option for infections caused by multidrug resistant Gram-negative pathogens, including *Pseudomonas aeruginosa*. Colistin resistance generally involves the modification of the lipid A moiety of lipopolysaccharide (LPS) with positively charged molecules, namely phosphoethanolamine (PEtN) or 4-amino-4-deoxy-L-arabinose (Ara4N), that reduce colistin affinity for its target. Several lines of evidence highlighted lipid A aminoarabinosylation as the primary colistin resistance mechanism in *P. aeruginosa*, while the contribution of phosphoethanolamination remains elusive. PEtN modification can be due to either endogenous (chromosomally encoded) PEtN transferase(s) (e.g., EptA in *P. aeruginosa*) or plasmid borne MCR enzymes, commonly found in enterobacteria. By individually cloning *eptA* and *mcr-1* into a plasmid for inducible gene expression, we demonstrated that MCR-1 and EptA have comparable PEtN transferase activity in *P. aeruginosa* and confer colistin resistance levels similar to those provided by lipid A aminoarabinosylation. Notably, EptA, but not MCR-1, negatively affects *P. aeruginosa* growth and, to a lesser extent, cell envelope integrity when expressed at high levels. Mutagenesis experiments revealed that PEtN transferase activity does not account for the noxious effects of EptA overexpression, that instead requires a C-terminal tail unique to *P. aeruginosa* EptA, whose function remains unknown. Overall, this study shows that both endogenous and exogenous PEtN transferases can promote colistin resistance in *P. aeruginosa*, and that PEtN and MCR-1 mediated resistance has no impact on growth and cell envelope homeostasis, suggesting that there may be no fitness barriers to the spread of *mcr-1* in *P. aeruginosa*.

## Introduction

The world is currently facing a worrying threat due to the spread of multidrug resistance in several bacterial pathogens. Emergence of resistance to most available antibiotics, including carbapenems, aminoglycosides, and fluoroquinolones, is increasing worldwide at an alarming rate, especially in Gram-negative bacteria, such as *Pseudomonas aeruginosa*, *Acinetobacter baumannii*, and Enterobacteriaceae (Theuretzbacher et al., 2020). The lack of new antimicrobials active against critical Gram-negative pathogens has prompted the medical community to re-evaluate the use of the old polymyxin antibiotic colistin (polymyxin E) in clinical practice when no other less toxic or effective antibiotics are available, and colistin is now considered a last-resort treatment option to combat recalcitrant Gram-negative infections (Falagas and Kopterides, 2007; Pogue et al., 2017; Nang et al., 2021).

Structurally, colistin is a decapeptide, consisting of a seven-member cyclic ring and a tripeptide side chain bound to a short fatty acid (Velkov et al., 2010). Its antibacterial activity relies on the interaction with the anionic lipid A moiety of lipopolysaccharide (LPS) in the outer membrane (OM) of Gram-negatives, leading to displacement of Ca^2+^ and Mg^2+^ cations that stabilize the LPS layer and, thus, causing derangement of the OM. This results in increased membrane permeability that allows colistin entry, leakage of cell contents and, ultimately, cell death (Poirel et al., 2017). Recent studies showed that the direct interaction of colistin with nascent LPS in the periplasmic leaflet of the inner membrane (IM) is crucial for its bacteriolytic activity (Sabnis et al., 2021).

Most Gram-negative bacteria acquire colistin resistance through genomic mutations in regulatory genes leading to induction of genes responsible for LPS remodeling, mostly through the covalent addition to lipid A of positively charge molecules, such as 4-amino-4-deoxy-L-arabinose (Ara4N) or phosphoethanolamine (PEtN), or by the acquisition of plasmid-borne PEtN transferase genes of the MCR family (Liu et al., 2016; Zhang et al., 2019). The resulting positive charge reduces LPS affinity for colistin, ultimately causing resistance (Olaitan et al., 2014; Jeannot et al., 2017; Moffatt et al., 2019; Sabnis et al., 2021).

*Pseudomonas aeruginosa* is a Gram-negative pathogen responsible for severe infections in immunocompromised patients and represents a major threat to cystic fibrosis (CF) patients. A hallmark of *P. aeruginosa* infections is the life-threatening severity and poor responsiveness to antibiotics due to intrinsic and acquired resistance (Poole, 2011). Colistin is currently used for treating multidrug-resistant *P. aeruginosa* infections, especially in CF (Langan et al., 2015). This, however, has inevitably led to the emergence of colistin-resistant *P. aeruginosa* strains (Falagas et al., 2010; Giamarellou, 2016), even though colistin resistance rates are overall lower as compared to other anti-pseudomonal agents (Horcajada et al., 2019; Gajdács et al., 2021).

In *P. aeruginosa* clinical isolates, colistin resistance is always associated with mutations leading to the overexpression of the *arn* operon, which encodes the enzymes for Ara4N modification of lipid A (Barrow and Kwon, 2009; Schurek et al., 2009; Moskowitz et al., 2012; Jochumsen et al., 2016; Jeannot et al., 2017). Accordingly, different studies have found that a functional Ara4N pathway is required for the acquisition of colistin resistance by *P. aeruginosa* in *in vitro* evolution experiments (Jochumsen et al., 2016; Chung et al., 2017; Lo Sciuto and Imperi, 2018), implying that lipid A aminoarabinosylation could represent a possible target for the development of anti-*Pseudomonas* colistin adjuvants. Indeed, inhibitors of the Ara4N transferase ArnT have been recently demonstrated to potentiate colistin activity against colistin-resistant *P. aeruginosa* strains (Ghirga et al., 2020; Quaglio et al., 2020).

In contrast, the role of PEtN modification of lipid A in *P. aeruginosa* colistin resistance is much less clear. The *P. aeruginosa* genome contains a functional ortholog of the PEtN transferase gene *eptA*, whose expression is induced by extracellular zinc through the two-component system ColR–ColS (Nowicki et al., 2015). Notably, zinc-induced expression of EptA and the consequent addition of PEtN to lipid A were, however, reported not to affect colistin MIC (Nowicki et al., 2015), leading to hypothesize that EptA-mediated lipid A phosphoethanolamination does not confer colistin resistance in *P. aeruginosa*. On the other hand, more recently, a couple of studies investigated the effect of the exogenous PEtN transferase MCR-1 on *P. aeruginosa* colistin resistance, by introducing engineered or native plasmids carrying *mcr-1* with its native promoter. PEtN modification of lipid A was invariably observed in MCR-1 expressing strains and conferred colistin resistance, even though the resistance level varied significantly among different isolates and was overall much lower than that observed in other Gram-negative pathogens, such as *Escherichia coli*, *Klebsiella pneumoniae*, or *A. baumannii* (Liu et al., 2016, 2017). Interestingly, whilst until recently *mcr* plasmids were mainly found in Enterobacteriaceae, in the last couple of years some reports described the sporadic isolation of *mcr*-positive colistin resistant isolates of *P. aeruginosa* (Hameed et al., 2019; Abd El-Baky et al., 2020; Tahmasebi et al., 2020a,b; Nitz et al., 2021). Although these studies did not provide genetic and/or molecular evidence that *mcr* genes were indeed responsible for the colistin resistance phenotype, this body of evidence suggests that lipid A phosphoethanolamination could play a role in colistin resistance also in *P. aeruginosa* and, thus, that the contribution of endogenous and exogenous PEtN transferases to colistin resistance might be different in this bacterium.

To clarify the role of PEtN modification of lipid A in colistin resistance and to directly compare the *in vivo* activities of endogenous (EptA) and exogenous (MCR-1) lipid A PEtN transferases in *P. aeruginosa*, in this work we cloned the *eptA* and *mcr-1* genes in a plasmid for isopropyl-β-D-thiogalactoside (IPTG)-inducible gene expression and analyzed the effect of ectopic EptA or MCR-1 expression on *P. aeruginosa* colistin resistance and fitness.

## Materials and Methods

### Bacterial Strains and Culture Media

Strains and plasmids are listed in Supplementary Table 1. Lysogeny broth (LB, Acumedia) was used for genetic manipulation. Mueller–Hinton broth (MH, Difco) was used for Kirby–Bauer assay, while cation-adjusted MH (MH II, Difco) was used for all the other assays. When required, IPTG was added at the indicated concentrations. All chemicals were purchased from Merck.

### Generation of Plasmids

To obtain the constructs for IPTG-inducible expression of *mcr-1*, *eptA*, or the truncated variant *eptA*^ΔC–ter^, the coding sequence of each gene was amplified by PCR using the genomic DNA of *P. aeruginosa* PAO1 (for *eptA* and *eptA*^ΔC–ter^) or the plasmid pHNSHP45 (for *mcr-1*) as the template (Supplementary Table 1). The amplicons were digested with the restriction enzymes *Eco*RI and *Xho*I and individually cloned into the sequencing plasmid pBlueScript II KS (pBS; Stratagene), previously digested with the same enzymes. The construct pBS*eptA* was used as template for PCR-mediated site-specific mutagenesis using the “Q5 Site-Directed Mutagenesis” kit (New England BioLabs), according to manufacturer’s instructions, and a primer pair specifically designed to introduce an A-to-G substitution leading to the mutation T278A, thus obtaining the *eptA*^T278A^ allele (Supplementary Table 1). After verification of the cloned fragments through restriction analysis and DNA sequencing, the inserts were excised from pBS and sub-cloned, using the same enzymes, into the shuttle vector pME6032, which replicates in *P. aeruginosa* and allows IPTG-inducible expression of cloned genes (Heeb and Haas, 2001). Primers used for cloning, site-specific mutagenesis and sequencing are listed in Supplementary Table 2. The constructs were transferred into *P. aeruginosa* strains by transformation.

### Gene Expression Analysis

The expression level of *eptA* was determined by quantitative reverse transcription PCR (qRT-PCR). Bacteria were cultured in MH II in the presence of increasing concentrations of IPTG until mid-log phase, harvested by centrifugation and treated with RNAprotect Bacteria Reagent (Qiagen). RNA was purified using the RNeasy Mini Kit (Qiagen), treated with DNase, and re-purified with the RNeasy MinElute Cleanup Kit (Qiagen). cDNA was reverse transcribed from 0.5 μg of RNA with Prime Script RT Reagent Kit (Takara). Appropriate dilutions of the cDNA were used as template for qRT-PCR in a AriaMx Real-Time PCR System (Agilent) using TB Green Premier EX Taq master mix (Takara) and the primers listed in Supplementary Table 2. Relative gene expression with respect to the housekeeping gene *rpoD* was calculated using the 2^–ΔΔ^ ^Ct^ method (Livak and Schmittgen, 2001).

### Lipid A Analysis

Bacteria were cultured in MH II until early stationary phase, harvested by centrifugation and lipid A was extracted using the ammonium hydroxide-isobutyric acid-based procedure (Liu et al., 2017) with previously described modifications (Lo Sciuto et al., 2018). Samples were analyzed by matrix-assisted laser desorption-time of flight (MALDI-TOF) in the negative-ion mode (Lo Sciuto et al., 2018).

### Growth Assays

Growth assays were performed in MH II in 96-well microtiter plates at 37°C (200 μL per well). Growth was measured as optical density at 600 nm (OD_600_) in a Tecan Spark 10M microtiter plate reader.

Pairwise competition experiments were performed by inoculating each strain of the pair of interest at 5 × 10^5^ cells/mL in MH II. After 16 and 20 h at 37°C, cultures were serially diluted in saline and plated onto MH II agar and MH II agar containing 2 μg/mL colistin and 0.5 mM IPTG, to evaluate the number of cells carrying pME*eptA* or pME*mcr-1* (that develop colonies both in the presence and absence of colistin) and the number of cells carrying the empty plasmid pME6032 (that develop colonies only in the absence of colistin). Preliminary experiments were performed to select the lowest colistin concentration that inhibits the growth of cells with the empty vector without affecting the plating efficiency of those carrying pME*eptA* or pME*mcr-1*. The competitive index was calculated as the ratio between the number of cells expressing EptA or MCR-1 and the number of cells carrying pME6032 at 16 or 24 h divided by to same ratio at time 0 (Lo Sciuto et al., 2020).

### MIC Assays

Colistin MIC was determined through the broth microdilution method in MH II. Strains were cultured at 37°C for 8 h, and then refreshed at 5 × 10^5^ cells/mL in the presence of increasing concentrations of colistin (up to 128 μg/mL). MIC was recorded after 24 h at 37°C. Each strain was tested in at least six independent experiments.

### Kirby–Bauer Disc Diffusion Assay

Bacterial cell suspensions in saline were normalized at 0.5 McFarland standard and swabbed onto MH agar plates. Disks containing ciprofloxacin (5 μg), imipenem (10 μg), gentamicin (10 μg), novobiocin (30 μg), erythromycin (15 μg), rifampicin (5 μg), or tobramycin (10 μg) (Becton Dickinson) were placed on the surface of the inoculated plates, and growth inhibition halo diameters were measured after 24-h incubation at 37°C.

### Detergent Sensitivity Assay

Bacteria were cultured in MH II until mid-exponential phase, harvested by centrifugation, resuspended in saline at OD_600_ of 0.5 and dispensed in microtiter plate wells in the presence of increasing concentrations (0–5%) of sodium dodecyl sulphate (SDS) (200 μL per well). Sensitivity to the SDS lytic effect was assessed by determining the turbidity (OD_600_) of cell suspensions after 5-min incubation at room temperature (Lo Sciuto et al., 2014), using a Tecan Spark 10M microtiter plate reader.

### Fluorescent Probe-Permeability Assays

Permeability assays were performed as previously described (Hancock and Farmer, 1993; Kwon et al., 2019), with few modifications. Bacteria were cultured in MH II until mid-exponential phase, harvested by centrifugation, resuspended in 5 mM HEPES (pH 7.2) at OD_600_ of 2 and dispensed in microtiter plate wells in the presence of 1-*N*-phenylnaphthylamine (NPN) or propidium iodide (PI) at 10 μM or 20 μg/mL final concentration, respectively. Fluorescence was measured in a Tecan Spark 10M microtiter plate reader (excitation at 350 nm and emission at 420 nm for NPN; excitation at 580 nm and emission at 620 nm for PI) after 5 min at room temperature, subtracted of the background values of samples without NPN or PI, and normalized to the OD_600_ of the cell suspension.

### *In silico* Analyses

Sequence alignments were generated with Clustal Omega.^1^ Residue conservation analysis was performed with the ConSurf server.^2^ The TMHMM server was used for transmembrane helix prediction.^3^ Three-dimensional modeling was performed with SWISS-MODEL,^4^ using the *Neisseria meningitidis* EptA protein (PDB 5FGN) as the template.

### Statistical Analyses

Statistical analysis was performed with GraphPad InStat, using one-way analysis of variance (ANOVA) followed by Tukey–Kramer multiple comparison test.

## Results

### Effect of EptA and MCR-1 on Lipid A

To investigate the contribution of PEtN-modification of the lipid A moiety of LPS to colistin resistance in *P. aeruginosa*, and to evaluate the effect of this lipid A modification on bacterial fitness, two plasmids were generated for the IPTG-inducible expression of either the endogenous lipid A PEtN transferase EptA or the exogenous analog MCR-1. These plasmids, named pME*eptA* and pME*mcr-1*, were individually introduced in two *P. aeruginosa* reference strains, namely PAO1 and PA14, that are distantly related and are representative of the two major strain groups of the *P. aeruginosa* population structure (Freschi et al., 2015).

**FIGURE 1:**
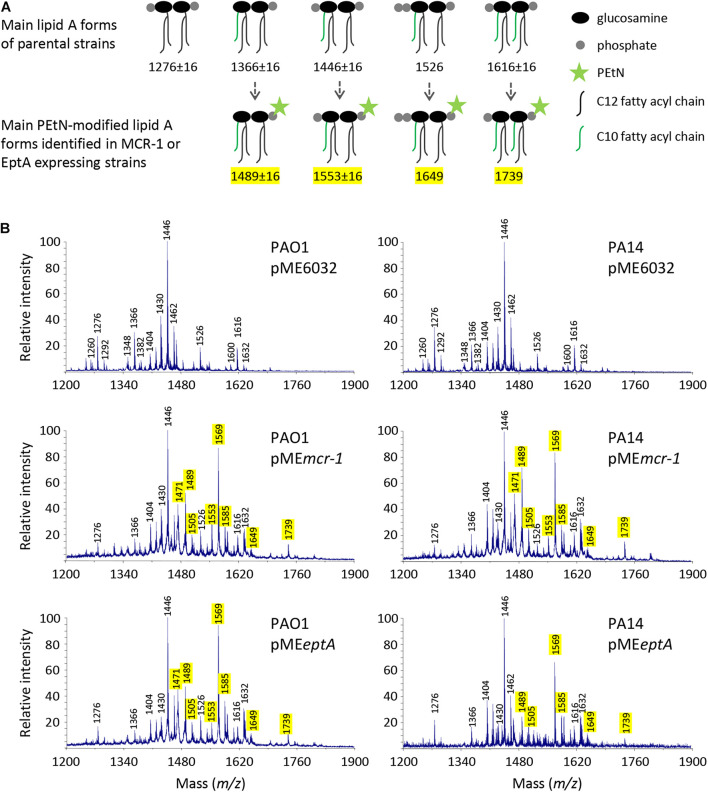
Effect of MCR-1 or EptA expression on lipid A in *P. aeruginosa* PAO1 and PA14. **(A)** Cartoons showing the relevant lipid A forms identified by mass spectrometry. **(B)** MALDI-TOF spectra of lipid A extracted from cells cultured in the presence of 0.5 mM IPTG. Spectra were obtained in the ion negative mode, so m/z values correspond to (molecular mass – 1)/1. m/z = 1276, diphosphorylated tetra-acylated lipid A; m/z = 1366, monophosphorylated penta-acylated lipid A; m/z = 1446, diphosphorylated penta-acylated lipid A; m/z = 1526, triphosphorylated penta-acylated lipid A; and m/z = 1616, diphosphorylated hexa-acylated lipid A. m/z + 123 indicates the addition of a PEtN group, while m/z ± 16 indicates lipid A forms that vary for the presence/absence of a hydroxyl group in the secondary C12 acyl chains (Nowicki et al., 2015; Liu et al., 2017; Lo Sciuto et al., 2019). The peak at m/z = 1471 likely corresponds to the phosphoethanolaminated form of a still non-identified lipid A species at m/z = 1348 (Ghirga et al., 2020; Lo Sciuto et al., 2020). The m/z values of peaks corresponding to the phosphoethanolaminated lipid A forms here identified are highlighted in yellow.

To confirm that phosphoethanolamination of lipid A occurs in the strains expressing MCR-1 or EptA, the lipid A was extracted from PAO1 and PA14 carrying the empty vector pME6032, pME*eptA*, or pME*mcr-1* cultured in the presence of 0.5 mM IPTG. Lipid A was then analyzed by MALDI-TOF mass spectrometry (Figure 1). The lipid A spectra were characterized by a major peak corresponding to diphosphorylated penta-acylated lipid A (m/z 1446), that represents the main lipid A form of laboratory *P. aeruginosa* strains (Lo Sciuto et al., 2018, 2020). Less abundant peaks were also identified, such as tetra-acylated diphosphorylated lipid A (m/z 1276), penta-acylated monophosphorylated lipid A (m/z 1366), penta-acylated triphosphorylated lipid A (m/z 1526), and hexa-acylated lipid A (m/z 1616). Notably, some lipid A forms were found with different hydroxylation states, due to the presence/absence of a hydroxyl group in the two secondary C12 acyl chains (Lo Sciuto et al., 2019).

As expected, additional peaks were exclusively detected in the lipid A spectra of cells carrying pME*eptA* or pME*mcr-1*. The addition of a PEtN group results in a + 123 shift in the m/z value of lipid A peaks (Nowicki et al., 2015; Liu et al., 2017). The peaks at m/z 1489 and 1569 correspond to the phosphoethanolaminated penta-acylated mono- or diphosphorylated form, respectively, while the peak at m/z 1739 corresponds to the phosphoethanolaminated hexa-acylated diphosphorylated form. Overall, lipid A analysis confirmed that both MCR-1 and EptA expressed from the pME*mcr-1*/*eptA* constructs are functional and that they efficiently modify lipid A with PEtN. Notably, PEtN-modified lipid A was not observed in both PAO1 and PA14 carrying the empty plasmid (Figure 1), confirming that the endogenous copy of *eptA*, that is present in the genome of both strains,^5^ is not or only poorly expressed in standard laboratory media (Nowicki et al., 2015; Liu et al., 2017; Lo Sciuto et al., 2018).

### Effect of Lipid A Phosphoethanolamination on Colistin Resistance

To investigate the effect of MCR-1 or EptA expression on colistin resistance, MIC assays were performed in the presence of different IPTG concentrations (Table 1). As expected, strains carrying the empty plasmid were susceptible to colistin, as inferred from MIC values lower than the clinical breakpoint for *P. aeruginosa* (2 μg/mL; EUCAST). *P. aeruginosa* expressing MCR-1 showed an IPTG-dose dependent increase in colistin MIC, confirming the capability of MCR-1 mediated lipid A phosphoethanolamination to confer colistin resistance in *P. aeruginosa* (Liu et al., 2017). Notably, colistin MIC was fourfold higher for PA14 pME*mcr-1* than for PAO1 pME*mcr-1* (Table 1). This is in line with the results of a recent study about the contribution of Ara4N-modified lipid A to colistin resistance, that was found to be significantly greater in PA14 with respect to PAO1 (Lo Sciuto et al., 2020). These results confirm previous evidence that PA14 is more responsive than PAO1 to lipid A modifications that confer colistin resistance (Gutu et al., 2015). Notably, when colistin MIC was directly compared between MCR-1 expressing strains, cultured in the presence of 0.5 mM IPTG, and recombinant strains that constitutively express the *arn* operon responsible for Ara4N modification of lipid A (PAO1/PA14 P*rpsA*:*arn*), in which the *arn* promoter was replaced with the promoter of the housekeeping gene *rpsA* (Lo Sciuto et al., 2020; Supplementary Table 1), it appeared that lipid A phosphoethanolamination is as efficient as aminoarabinosylation in conferring colistin resistance to *P. aeruginosa* (Table 1).

**TABLE 1:** MIC of colistin for *P. aeruginosa* PAO1 and PA14 expressing or not MCR-1, EptA or EptA variants.

Strain	Colistin MIC (μg/mL)^a^ with IPTG at		
	0 mM	0.125 mM	0.5 mM
PAO1 pME6032	1	1	1
PAO1 pME*mcr-1*	2	8	8
PAO1 pME*eptA*	4	2	1
PAO1 pME*eptA*^T278A^	NT	1	1
PAO1 pME*eptA*^ΔC–ter^	NT	4	4
PAO1 P*rpsA*:*arn*^b^	8	NT	NT
PA14 pME6032	1	1	0.5
PA14 pME*mcr-1*	1	16	32
PA14 pME*eptA*	8	16	2
PA14 pME*eptA*^T278A^	NT	1	0.5
PA14 pME*eptA*^ΔC–ter^	NT	8	8
PA14 P*rpsA*::*arn*^b^	32	NT	NT

*^*a*^Values correspond to the mode of at least six experiments. NT, not tested.*

*^*b*^These recombinant strains constitutively express the *arn* operon and have Ara4N-modified lipid A (Lo Sciuto et al., 2020).*

**FIGURE 2:**
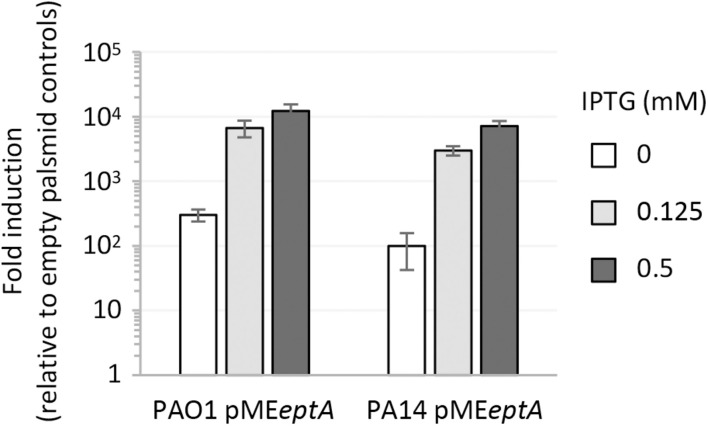
Expression level of *eptA*, determined by qRT-PCR, in *P. aeruginosa* PAO1 or PA14 carrying pME*eptA* cultured in the presence of increasing IPTG concentrations. Relative *eptA* gene expression is shown as fold induction with respect to the cognate strain carrying the empty plasmid pME6032. Data represent the mean (±SD) of two independent experiments performed in triplicate.

In contrast, colistin resistance in EptA overexpressing strains did not correlate with the IPTG concentration in the medium. Indeed, colistin MIC was higher at 0 (for PAO1) or 0.125 mM IPTG (for PA14) and reverted at levels comparable to the empty plasmid controls at 0.5 mM IPTG (Table 1). The significant increase in colistin MIC observed in strains carrying pME*eptA* under non-inducing conditions prompted us to verify lipid A phosphoethanolamination and *eptA* expression levels at different IPTG concentrations. Mass spectrometry analysis revealed that lipid A was efficiently phosphoethanolaminated even in the absence of IPTG (Supplementary Figure 1). This is in line with the results of the qRT-PCR analysis, which showed that *eptA* expression was hundreds-fold higher in pME*eptA* carrying strains under non-inducing conditions as compared to empty plasmid controls (Figure 2). Overall, these experiments revealed that *P. aeruginosa* EptA can promote colistin resistance at levels almost comparable to those conferred by the exogenous PEtN transferase MCR-1, even if proper EptA levels seem to be critical for optimal induction of resistance.

### Effect of EptA and MCR-1 on Bacterial Fitness

**FIGURE 3:**
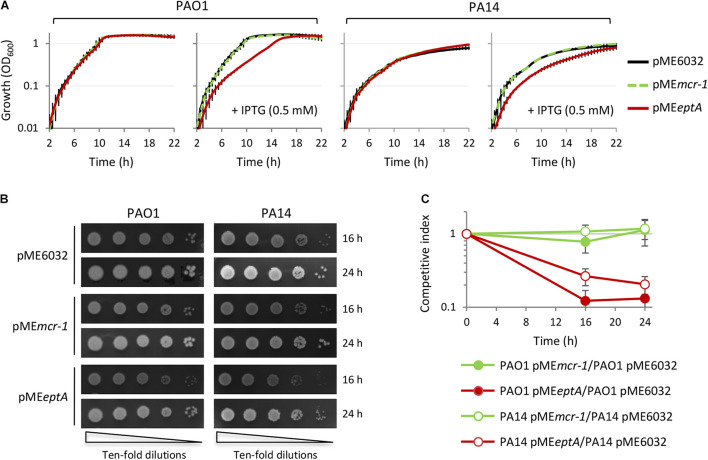
Effect of MCR-1 or EptA overexpression on *P. aeruginosa* growth. **(A)** Growth curves of PAO1 or PA14 containing the empty vector pME6032 (black lines), pME*mcr-1* (green lines), or pME*eptA* (red lines), cultured in the absence or in the presence of 0.5 mM IPTG. **(B)** Colony growth of the same strains described in **(A)** on agar plates containing 0.5 mM IPTG. **(C)** *In vitro* competition between PAO1 or PA14 cells with the empty vector and cells overexpressing MCR-1 or EptA in the presence of 0.5 mM IPTG. Data represent the mean (±SD) or are representative of at least three independent experiments.

The relevant decrease in colistin MIC observed in EptA expressing cells cultured in the presence of 0.5 mM IPTG (Table 1) led us to hypothesize that EptA could exert some toxicity when expressed at very high levels (about 10,000-fold higher than the expression level of the empty plasmid controls; Figure 2), that could somehow counterbalance the positive effect of PEtN-modified lipid A on colistin resistance. To evaluate whether EptA overexpression causes any fitness costs, we compared planktonic and colony growth between EptA/MCR-1 expressing cells and the empty plasmid controls. As shown in Figure 3A and Supplementary Figure 2, MCR-1 expression did not affect growth of any strain at any IPTG concentration. In contrast, the expression of EptA caused a reduction in the growth kinetics of both PAO1 and PA14 in an IPTG-dose dependent manner, with maximum growth inhibition observed at IPTG concentrations ≥0.5 mM (Figure 3A and Supplementary Figure 2). Colony growth over time was also slightly delayed for EptA overexpressing strains as compared to the other strains (Figure 3B), corroborating the negative effect of EptA on *P. aeruginosa* growth kinetics. The growth defects of EptA overexpressing cells were further investigated through competition experiments, by co-culturing cells carrying pME*eptA* (or pME*mcr-1* as the control) with those with the empty vector in the presence of 0.5 mM IPTG. The results are shown as competitive index, representing the relative growth of EptA (or MCR-1) overexpressing strains with respect to the empty plasmid controls (Figure 3C). In agreement with the results of mono-culture assays, cells expressing MCR-1 showed a competitive index close to 1, indicating that their growth kinetic was overall comparable to that of cells lacking *mcr-1*. On the other hand, both PAO1 pME*eptA* and PA14 pME*eptA* showed a much lower competitive index (0.1 and 0.2, respectively), implying that EptA overexpressing cells were outcompeted by cells harboring the empty plasmid.

**FIGURE 4:**
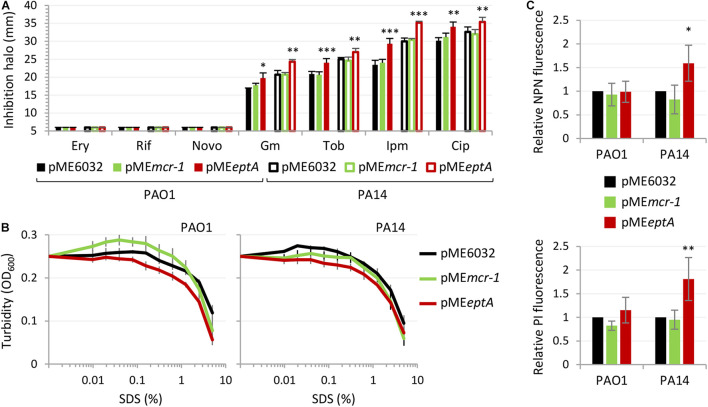
Effect of MCR-1 or EptA overexpression on antibiotic sensitivity and cell envelope integrity. **(A)** Inhibition halos in the Kirby–Bauer disc diffusion assay of erythromycin (Ery), rifampicin (Rif) novobiocin (Novo), gentamicin (Gm), tobramycin (Tob), imipenem (Ipm), or ciprofloxacin (Cip) for PAO1 or PA14 containing pME6032, pME*mcr-1*, or pME*eptA*. **(B)** Lytic effect of SDS, measured as decrease in cell suspension turbidity (OD_600_), on PAO1 or PA14 cells containing pME6032, pME*mcr-1*, or pME*eptA*. **(C)** Uptake of NPN (upper panel) and PI (lower panel) by PAO1 or PA14 cells containing pME6032, pME*mcr-1*, or pME*eptA*, reported as fluorescence emission relative to the empty plasmid controls. In all the assays, strains were cultured in the presence of 0.5 mM IPTG. Data represent the mean (±SD) of three **(A,B)** or five **(C)** independent experiments. Asterisks indicate a statistically significant difference with respect to the corresponding empty plasmid control (^∗^*P* < 0.05, ^∗∗^*P* < 0.01, ^∗∗∗^*P* < 0.001; ANOVA).

To investigate whether the detrimental effect EptA overexpression on *P. aeruginosa* growth correlated with defects in the integrity and/or permeability barrier of the cell envelope, we compared the sensitivity profile to different antibiotics belonging to different classes of *P. aeruginosa* PAO1 and PA14 carrying the empty plasmid pME6032 or the pME*mcr-1*/pME*eptA* derivatives cultured in the presence of 0.5 mM IPTG, by using the Kirby–Bauer disc diffusion test. MCR-1 expressing strains did not show any difference in the resistance profile with respect to the empty plasmid controls, while EptA overexpressing strains showed a slight increase in susceptibility to some antibiotics, i.e., ciprofloxacin, gentamicin, tobramycin, and imipenem (Figure 4A). All strains remained completely insensitive to high molecular weight antibiotics, such as novobiocin, erythromycin, and rifampicin, that are poorly active against *P. aeruginosa* because of poor diffusion across the OM and that are generally used to highlight defects in the OM integrity (Nikaido, 2005; Lo Sciuto et al., 2014, 2018). This suggests that EptA overexpressing cells have minor defects (if any) in cell envelope homeostasis. This was confirmed by the analysis of the sensitivity profile to the lytic effect of SDS, that was overall comparable between MCR-1/EptA overexpressing cells and the empty plasmid controls (Figure 4B). To further investigate the possible effect of PEtN and/or PEtN transferases on the *P. aeruginosa* cell envelope, the OM and IM permeability was assessed through the fluorescent probes NPN and PI, respectively (Hancock and Farmer, 1993; Kwon et al., 2019; Figure 4C). No differences were observed in NPN and PI fluorescence between MCR-1 expressing cells and empty plasmid controls. In contrast, EptA overexpression slightly increased NPN and PI uptake in PA14 but not in PAO1, suggesting that EptA might have a strain-specific effect on OM and IM integrity. Notably, such increase in membrane permeability can hardly explain the observed growth defects of EptA expressing cells, as both mono- and co-culture assays revealed that the negative impact of EptA overexpression on growth is more severe in PAO1 than in PA14 (Figure 3).

Overall, these assays revealed that MCR-1 and lipid A phosphoethanolamination do not influence cell envelope homeostasis in *P. aeruginosa*, while EptA overexpression has a marginal effect on the cell envelope permeability barrier, which is, however, unrelated to the growth defects of EptA expressing cells.

### The Growth Inhibitory Effect of EptA Is Not Related to Phosphoethanolamine Transferase Activity

**FIGURE 5:**
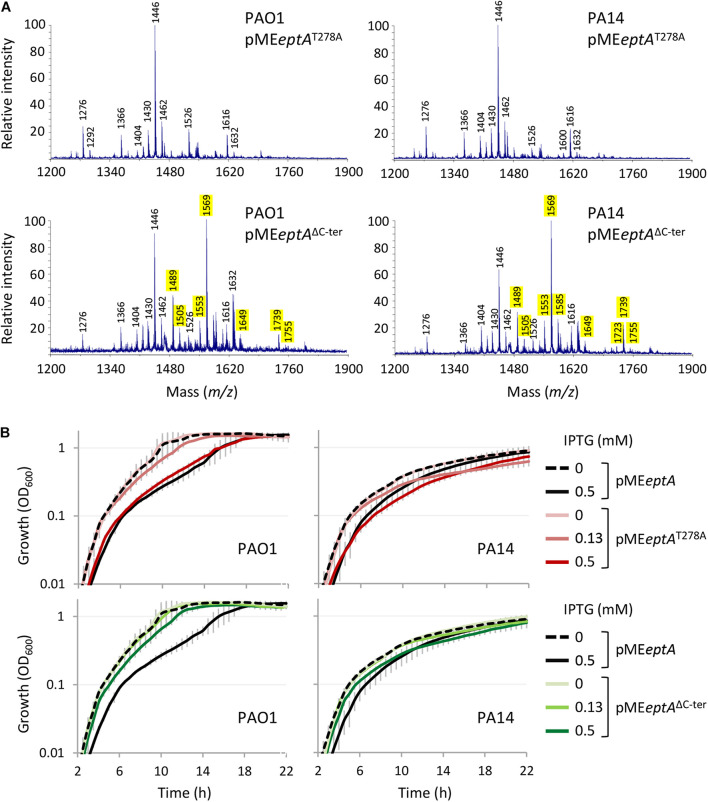
Effect of the expression of EptA variants mutated in a catalytic residue (EptA^T278A^) or deleted of 22 amino acids at the C-terminus (EptA^ΔC–ter^) in *P. aeruginosa*. **(A)** MALDI-TOF spectra of lipid A extracted from PAO1 or PA14 containing pME*eptA*, pME*eptA*^T278A^, or pME*eptA*^ΔC–ter^ cultured in the presence of 0.125 mM IPTG. The m/z values of peaks corresponding to phosphoethanolaminated lipid A forms are highlighted in yellow. See the legend to Figure 1 for further details. **(B)** Growth curves of PAO1 or PA14 containing pME*eptA*, pME*eptA*^T278A^, or pME*eptA*^ΔC–ter^ cultured in the presence of the indicated IPTG concentrations. Data represent the mean (±SD) or are representative of at least three independent experiments.

**FIGURE 6:**
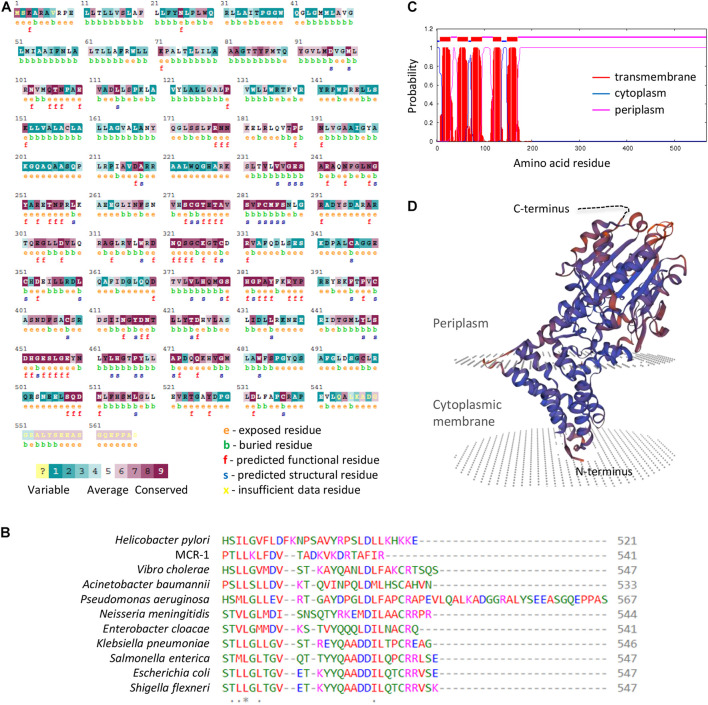
**(A)** ConSurf analysis of evolutionary residue conservation in *P. aeruginosa* EptA. **(B)** Alignment of the C-terminus of selected EptA orthologs from the indicated bacteria. The MCR-1 protein has been included as control. The corresponding full-length alignment is shown in Supplementary Figure 3. **(C)** Prediction of transmembrane helices in *P. aeruginosa* EptA by TMHMM. **(D)** Modeling of the *P. aeruginosa* EptA structure by SWISS-MODEL using the *N. meningitidis* EptA protein (PDB 5FGN) as the template. The C-terminal tail of *P. aeruginosa* EptA cannot be modeled and is represented as a dashed black line.

The finding that EptA but not MCR-1 overexpression is deleterious to *P. aeruginosa* strongly suggests that the growth defects might not be related to PEtN-modified lipid A. This, however, does not rule out that the PEtN transferase activity might be involved in the noxious effects of EptA overexpression. In fact, while most PEtN transferases have high substrate specificity (Zhang et al., 2019), a PEtN transferase of *Campylobacter jejuni* is more promiscuous, being able to add PEtN to (1) lipid A, (2) heptose I of the LPS inner core, (3) a Thr residue of the flagellar protein FlgG, and (4) the N-terminally linked glycans of some periplasmic proteins (Cullen et al., 2013). Considering that *P. aeruginosa* EptA has been poorly characterized so far (Nowicki et al., 2015), we attempted to verify the relevance of PEtN transferase activity for EptA-dependent toxicity. To this aim, we generated a construct (pME*eptA*^T278A^) to express a catalytically inactive EptA variant (EptA^T278A^), mutagenized in a conserved catalytic residue essential for *N. meningitidis* EptA activity (Xu et al., 2018a; Supplementary Figure 3). As expected, lipid A was not modified with PEtN in EptA^T278A^ overexpressing cells (Figure 5A and Supplementary Figure 4) and, accordingly, colistin MIC for these cells was identical to that for the empty plasmid controls (Table 1). Interestingly, overexpression of EptA^T278A^ caused growth defects comparable to those caused by wild type EptA (Figure 5B), indicating that PEtN transferase activity is not responsible for *P. aeruginosa* EptA deleterious effects. We therefore decided to search for possible unique feature(s) of *P. aeruginosa* EptA, by evaluating the evolutionary conservation among 150 representative EptA orthologs through the ConSurf server (Ashkenazy et al., 2016). This analysis highlighted a 25-aa C-terminal tail in the periplasmic domain of *P. aeruginosa* EptA that is absent from all other EptA orthologs (Figure 6). The IPTG-inducible expression of a truncated EptA variant lacking the last 22 amino acids (EptA^ΔC–ter^) in *P. aeruginosa* cells, followed by mass spectrometry analysis of lipid A, revealed that the C-terminal extension is not required for lipid A phosphoethanolamination (Figure 5A). In contrast, cells expressing the EptA^ΔC–ter^ variant showed less severe growth defects as compared to cells expressing wild type EptA (Figure 5B), implying that the C-terminal tail is partly responsible for the growth inhibitory effect of EptA overexpression. Moreover, EptA^ΔC–ter^ expressing strains showed high colistin MIC even in the presence of high IPTG concentrations (Table 1), supporting the hypothesis that the inverse proportion between wild type EptA expression and colistin resistance is likely due to the negative impact of high EptA levels on *P. aeruginosa* growth. Although previous studies have shown that also other PEtN transferases can negatively impact bacterial physiology (Xu et al., 2018b; Yang et al., 2020), this is the first report that associates PEtN transferase toxicity to a specific and evolutionary divergent protein domain. Further studies are required to elucidate the mechanistic basis of EptA-mediated toxicity in *P. aeruginosa*.

## Conclusion

While several studies highlighted the importance of lipid A aminoarabinosylation for colistin resistance in *P. aeruginosa*, the role of lipid A phosphoethanolamination in this bacterium remained elusive. Here, we have demonstrated that (1) exogenous and endogenous PEtN transferases (MCR-1 and EptA) have overall comparable lipid A PEtN transferase activity in *P. aeruginosa*, (2) both are effective in promoting colistin resistance, and (3) modification of lipid A with PEtN confers colistin resistance levels comparable to Ara4N. EptA overexpression was found to negatively impact *P. aeruginosa* fitness, in a manner that is independent of the lipid A phosphotransferase activity but dependent on the presence of a C-terminal extension specific to *P. aeruginosa* EptA. The mechanism by which this C-terminal tail affects growth has not been investigated in this study, but we are tempted to speculate that it could not be physiologically relevant, as growth defects were only observed at very high EptA expression levels, thousands-fold higher than the basal levels. For comparison, the only cue that has been proven to trigger *P. aeruginosa eptA* transcription to date (i.e., extracellular zinc) only caused a 20-fold increase in *eptA* gene expression (Nowicki et al., 2015).

Given the efficacy of EptA in conferring colistin resistance, the question arises as to why lipid A aminoarabinosylation is preferred over lipid A phosphoethanolamination as colistin resistance mechanism in *P. aeruginosa* during *in vitro* evolution experiments as well as in the clinical setting (Barrow and Kwon, 2009; Schurek et al., 2009; Moskowitz et al., 2012; Jochumsen et al., 2016; Chung et al., 2017; Lo Sciuto and Imperi, 2018). Even if we cannot exclude that the fitness cost associated with the EptA C-terminal domain could somehow favor Ara4N producing cells over PEtN producing ones, an alternative hypothesis is that the evolutionary pathways to Ara4N-mediated resistance are more likely to occur due to the high number of genes and, thus, of possible mutations directly or indirectly triggering *arn* gene expression (Barrow and Kwon, 2009; Schurek et al., 2009; Moskowitz et al., 2012; Jochumsen et al., 2016; Jeannot et al., 2017). Since very few is known about *eptA* regulation in *P. aeruginosa* (Nowicki et al., 2015), further studies are needed to decipher the regulatory circuit(s) controlling *eptA* expression and the likelihood of *eptA*-inducing mutations in *P. aeruginosa* and, therefore, to support or deny such hypothesis.

On the other hand, the present study has also shown that MCR-1 mediated colistin resistance in *P. aeruginosa* has no impact on relevant physiological aspects, such as growth, drug sensitivity and cell envelope integrity, suggesting that there would be no fitness barriers to the spread of *mcr* genes in the *P. aeruginosa* population. This is an issue that deserves further investigation, especially in view of the recent identification of *mcr* harboring *P. aeruginosa* clinical isolates in different geographic areas (Hameed et al., 2019; Abd El-Baky et al., 2020; Tahmasebi et al., 2020a,b; Nitz et al., 2021).

## Data Availability Statement

The original contributions presented in the study are included in the article/Supplementary Material, further inquiries can be directed to the corresponding author.

## Author Contributions

AL and FI conceived and designed the experiments. MC, AL, and CB performed the experiments. MC, AL, CM, and FI analyzed the data. CM and FI contributed reagents, materials, and analysis tools. FI wrote the manuscript. All authors read and approved the final manuscript.

## Conflict of Interest

The authors declare that the research was conducted in the absence of any commercial or financial relationships that could be construed as a potential conflict of interest.

## Publisher’s Note

All claims expressed in this article are solely those of the authors and do not necessarily represent those of their affiliated organizations, or those of the publisher, the editors and the reviewers. Any product that may be evaluated in this article, or claim that may be made by its manufacturer, is not guaranteed or endorsed by the publisher.

## Footnotes

1
https://www.ebi.ac.uk/Tools/msa/clustalo/

2
https://consurf.tau.ac.il/index_proteins.php

3
http://www.cbs.dtu.dk/services/TMHMM/

4
https://swissmodel.expasy.org/interactive

5
www.pseudomonas.com
