# Supplementary material for: Exogenous and Endogenous Phosphoethanolamine Transferases Differently Affect Colistin Resistance and Fitness in Pseudomonas aeruginosa
Source: Front Microbiol. 2021 Oct 27;12:778968. doi: 10.3389/fmicb.2021.778968 (PMC8578941; doi:10.3389/fmicb.2021.778968)
Supplement: Supplementary file 1 [file Data_Sheet_1.PDF]

frontiers

Supplementary Material

Exogenous and endogenous phosphoethanolamine transferases differently affect colistin resistance and fitness in *Pseudomonas aeruginosa*

Matteo Cervoni[1#], Alessandra Lo Sciuto[1#], Chiara Bianchini[1], Carmine Mancone[2], Francesco Imperi[1,3*]

[1] Department of Science, Roma Tre University, Rome, Italy

[2] Department of Molecular Medicine, Sapienza University of Rome, Rome, Italy

[3] IRCCS Fondazione Santa Lucia, Rome, Italy

[#]These authors contributed equally to the work.

*** Correspondence:** Francesco Imperi, francesco.imperi@uniroma3.it

1. Supplementary Figures and Tables

1.1. Supplementary Figures

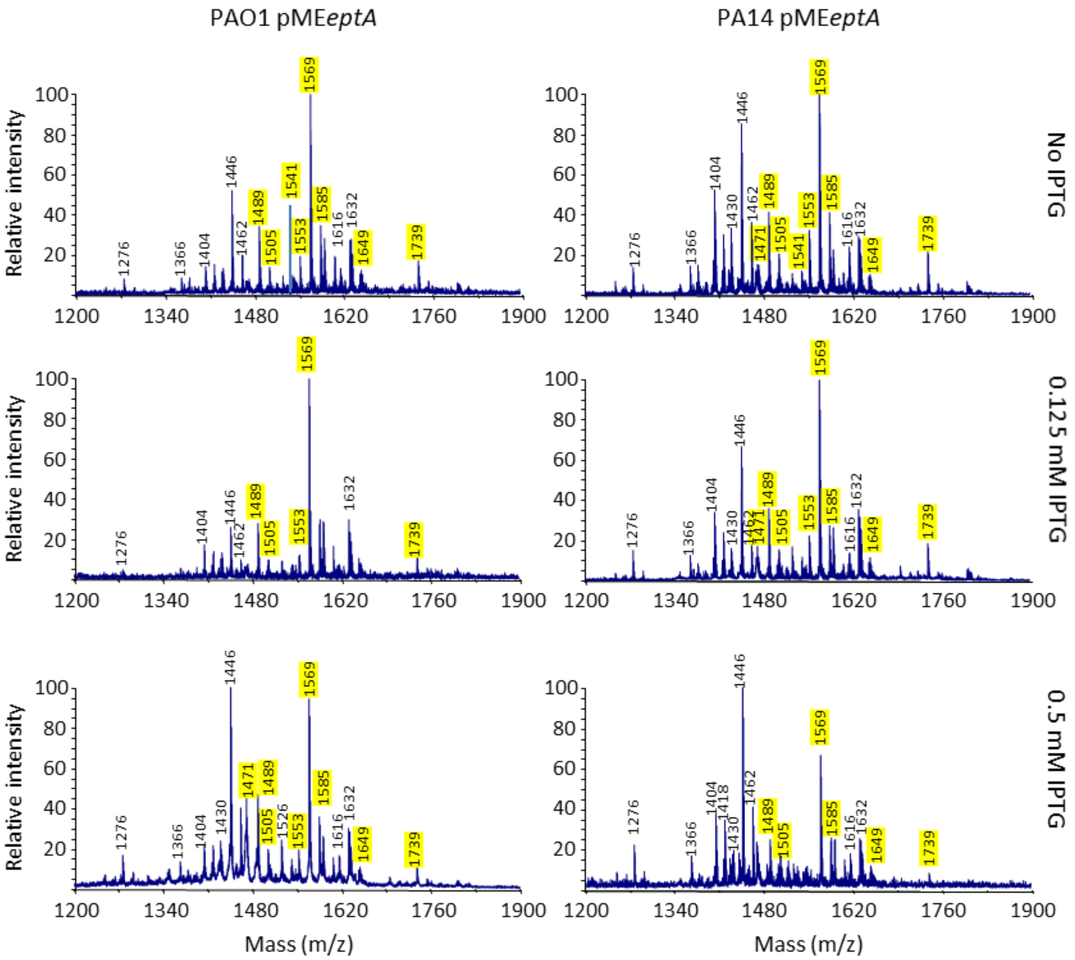

Supplementary Figure 1. MALDI-TOF spectra of lipid A extracted from PAO1 pME*eptA* or PA14 pME*eptA* cultured in the absence or presence of IPTG at 0.125 or 0.5 mM. The m/z values of peaks corresponding to phosphoethanolaminated lipid A forms are highlighted in yellow. Spectra are representative of three biological replicates giving similar results.

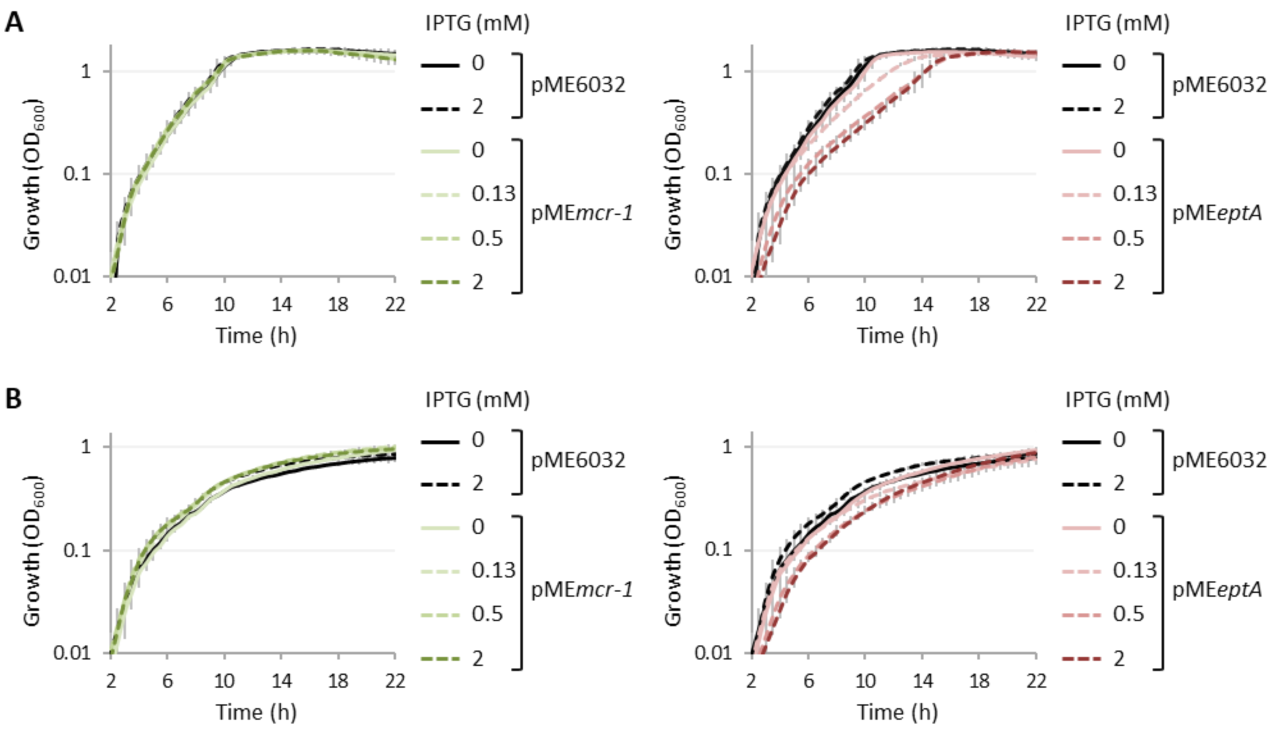

Supplementary Figure 2. Growth curves of (A) PAO1 or (B) PA14 carrying pME6032, pME*mcr-1* or pME*eptA* in the presence of increasing IPTG concentrations (0-2 mM). Data represent the mean (± standard deviation) of three independent experiments.

Supplementary Figure 3. Full-length alignment of selected EptA orthologs from *Acinetobacter baumannii* (SSI74383.1), *Enterobacter cloacae* (WP_059385803.1), *Escherichia coli* (OWW57050.1), *Helicobacter pylori* (WP_189394748.1), *Klebsiella pneumoniae* (CCI78008.1), *Neisseria meningitidis* (AKM91408.1), *Pseudomonas aeruginosa* (WP_003113468.1), *Salmonella enterica* (QNL54535.1), *Shigella flexneri* (QLG55136.1), *Vibrio cholerae* (TYC39410.1). The MCR-1 protein has been included as control (WP_049589868.1). The highly conserved catalytic residue mutated in the EptA^{T278A} variant and the 22 residues deleted in the EptA$^{\Delta C\text{-}ter}$ variant are highlighted with a black box and a red box, respectively.

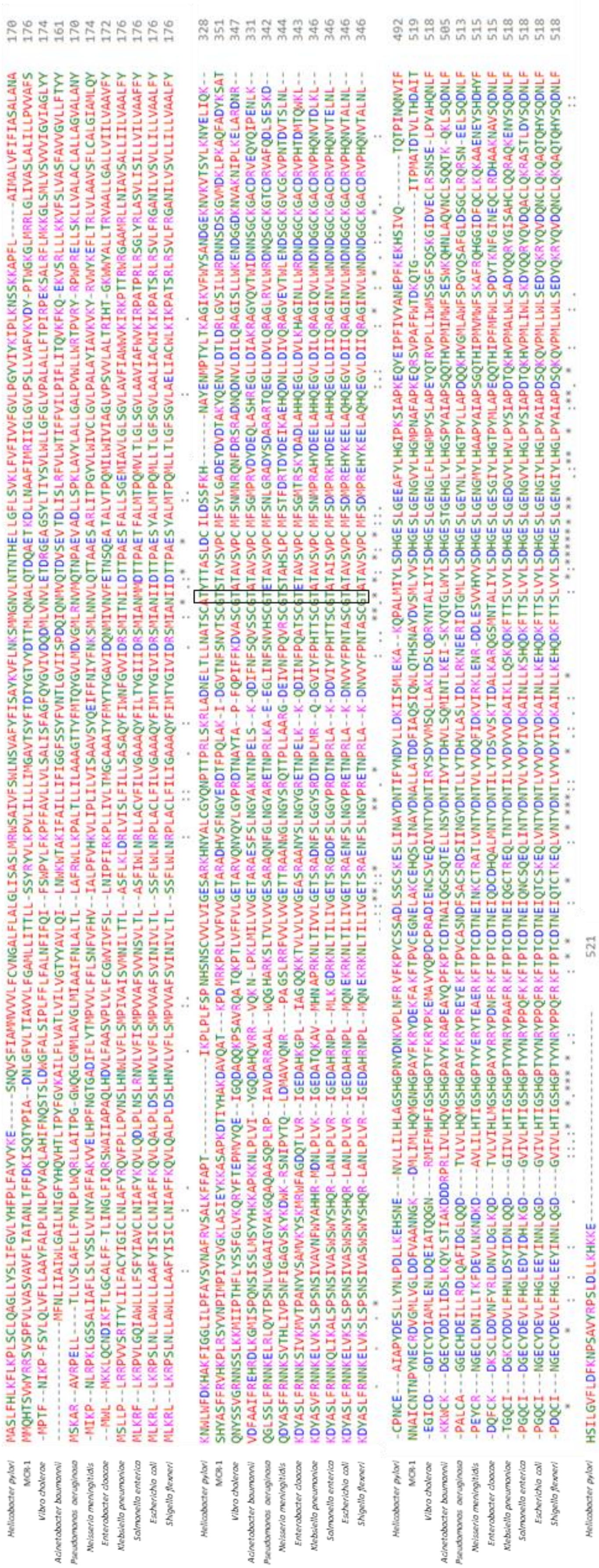
Helicobacter pylori
MCR-1
Vibrio cholerae
Acinetobacter baumannii
Pseudomonas aeruginosa
Neisseria meningitidis
Enterobacter cloacae
Klebsiella pneumoniae
Salmonella enterica
Escherichia coli
Shigella flexneri

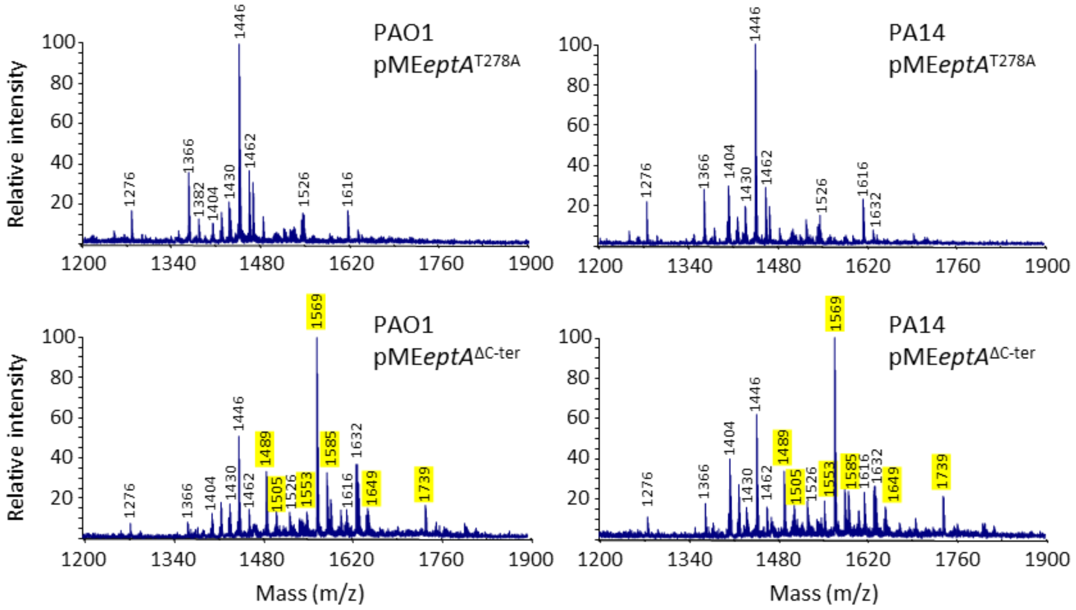

Supplementary Figure 4. MALDI-TOF spectra of lipid A extracted from PAO1 or PA14 cells expressing EptA variants mutated in a catalytic residue (EptA^{T278A}) or deleted of 22 amino acids at the C-terminus (EptA$^{\Delta C\text{-}ter}$) cultured in the presence of 0.5 mM IPTG. The m/z values of peaks corresponding to phosphoethanolaminated lipid A forms are highlighted in yellow. Spectra are representative of three biological replicates giving similar results.

1.2. Supplementary Table

Supplementary Table 1. Bacterial strains and plasmids used in this study.

Strain or plasmid	Genotype and/or relevant characteristics	Reference or source
P. aeruginosa		
PAO1 (ATCC15692)	Reference isolate	American type culture collection
PA14	Reference isolate	Rahme et al., 1995
PAO1 P*rpsA*::*arn*	PAO1 derivative in which the promoter of the *arn* operon is replaced by the promoter of the housekeeping gene *rpsA*	Lo Sciuto et al., 2020
PA14 P*rpsA*::*arn*	PA14 derivative in which the promoter of the *arn* operon is replaced by the promoter of the housekeeping gene *rpsA*	Lo Sciuto et al., 2020
E. coli		
DH5αF'	*recA1 endA1 hsdR17 supE44 thi-1 gyrA96 relA1* Δ(*lacZYA-argF*)U169[ϕ*80 dlacZ*Δ*M15*], Nal^R	Liss, 1987
**Plasmid**		
pHNSHP45	*mcr-1* carrying plasmid	Liu et al., 2016
pBluescript II KS (pBS)	Sequencing vector; ColE1 replicon; Ap^R	Stratagene
pBS*eptA*	pBS containing the coding sequence of the *eptA* gene from *P. aeruginosa* PAO1 (PA1972)	This study
pBS*mcr-1*	pBS containing the coding sequence of the *mcr-1* gene from plasmid pHNSHP45	This study
pBS$eptA^{T278A}$	pBS containing the *eptA* coding sequence with a point mutation in the codon 278 leading to a T278A substitution	This study
pBS$eptA^{\Delta C\text{-}ter}$	pBS containing a truncated *eptA* allele encoding for an EptA variant lacking the last 22 amino acids	This study
pME6032	IPTG-inducible expression vector, $lacI^Q$, Tc^R.	Heeb and Haas, 2001
pME*eptA*	pME6032 containing the *eptA* (PA1972) coding sequence from pBS*eptA* cloned downstream of the IPTG-inducible promoter	This study
pME*mcr-1*	pME6032 containing the *mcr-1* coding sequence from pBS*mcr-1* cloned downstream of the IPTG-inducible promoter	This study
pME$eptA^{T278A}$	pME6032 containing the $eptA^{T278A}$ allele from pBS$eptA^{T278A}$ cloned downstream of the IPTG-inducible promoter	This study
pME$eptA^{\Delta C\text{-}ter}$	pME6032 containing the $eptA^{\Delta C\text{-}ter}$ allele from pBS$eptA^{\Delta C\text{-}ter}$ cloned downstream of the IPTG-inducible promoter	This study

Supplementary Table 2. Primers used in this study.

**Primer name**	**Sequence (5'-3')**[1]	**Restriction sites**[2]	**Application**
*mcr-1*_pME6032_FW	cggaattcATGATGCAGCATACTTCTGTG	EcoRI	Generation of pBS*mcr-1*
*mcr-1*_pME6032_RV	ccctcgagTCAGCGGATGAATGCGGTG	XhoI	
*eptA*_pME6032_FW	cggaattCATGTCGAAAGCCCGCGC	EcoRI	Generation of pBS*eptA*
*eptA*_pME6032_RV	ccctcgaGTATCAGGAAGCCGGCGG	XhoI	
*eptA*_T278A_FW	CGGTACCGAGgCCGCGGTGTC		Generation of pBS*eptA*^{T278A}
*eptA*_T278A_RV	CAGGAGTGCACGTTGGAGAAGTTGATC		
*eptA*_Δ22aa_RV[3]	ccctcgagtcaCGCCTGCAGGACCTCGGG	XhoI	Generation of pBS*eptA*$^{\Delta C\text{-}ter}$
*eptA*_RT_FW[4]	TGCCCTGCATGTTCTCCAAC		qRT-PCR
*eptA*_RT_RV[4]	GATCCTTGCTCTCGCTCAGG		
*rpoD*_RT_FW	GGGCGAAGAAGGAAATGGTC		
*rpoD*_RT_RV	CAGGTGGCGTAGGTGGAGAA		
M13_FW	GTTTTCCCAGTCACGAC		DNA sequencing from pBS
M13_RV	AACAGCTATGACCATG		

[1] Lowercase letters indicate the region of the primer that does not anneal to the template.

[2] The restriction site used for cloning is underlined in the primer sequence.

[3] This primer was paired with *eptA*_pME6032_FW to amplify the truncated *eptA*$^{\Delta C\text{-}ter}$ variant.

[4] Primers described and used in Nowicki et al., 2015.

Additional references (not included in the main text)

Liss, L. (1987). New M13 host: DH5 F′ competent cells. *Focus* 9, 13.

Rahme, L.G., Stevens, E.J., Wolfort, S.F., Shao, J., Tompkins, R.G., Ausubel, F.M. (1995). Common virulence factors for bacterial pathogenicity in plants and animals. *Science* 268, 1899-1902.
